# A comparative, retrospective, observational study of the clinical and microbiological profiles of post-penetrating keratoplasty keratitis

**DOI:** 10.1038/srep32751

**Published:** 2016-09-02

**Authors:** I-Huang Lin, Yi-Sheng Chang, Sung-Huei Tseng, Yi-Hsun Huang

**Affiliations:** 1 Department of Ophthalmology, National Cheng Kung University Hospital, College of Medicine, National Cheng Kung University, Tainan, Taiwan; 2 Department of Ophthalmology, College of Medicine, National Cheng Kung University, Tainan, Taiwan; 3 Institute of Clinical Medicine, College of Medicine, National Cheng Kung University, Tainan, Taiwan

## Abstract

Infectious keratitis after penetrating keratoplasty (PK) is a devastating condition that may result in graft failure and poor visual outcome. In this study, we retrospectively reviewed the medical records of patients who underwent PK between 2009 and 2014, and recorded those who developed infectious keratitis. We compared the predisposing factors and organisms isolated to those identified in our previous study, conducted between 1989 and 1994. The incidence of post-PK infectious keratitis decreased from 11.6% (41 out of 354 cases, 1989–1994) to 6.5% (9 out of 138 cases, 2009–2014). Graft epithelial defect and suture-related problems remained the leading two risk factors of infectious keratitis after PK. Gram-positive and Gram-negative bacterial infection decreased from 58.5% and 46.3% to 11.1% and 22.2%, respectively (P = 0.023 and P = 0.271). In contrast, fungus infection increased from 9.8% to 66.7% (P = 0.001); fungi have become the major pathogen for post-PK infectious keratitis. In conclusion, while the incidence of post-PK infectious keratitis has decreased over time, the number and frequency of fungal infections have significantly increased in the recent study period. Clinicians should be aware of the shifting trend in pathogens involved in post-PK infectious keratitis.

Although the numbers of deep anterior lamellar keratoplasty (DALK) and endothelial keratoplasty (EK) performed have increased in recent years, penetrating keratoplasty (PK) probably remains the most common keratoplasty procedure performed worldwide^1^. In the past few decades, many improvements in microscopic instruments and surgical techniques used for PK have been made; however, microbial keratitis after PK is still a devastating problem that may result in graft failure and poor visual outcome^2^. During the past 20 years, the incidence of infectious keratitis after PK ranged from 1.76% to 12.1%^3^,^4^,^5^,^6^,^7^. In our previous study, the precipitating factors for post-PK infectious keratitis included epithelial defects, suture-related problems, use of contact lenses, trichiasis, dry eye, and lid abnormalities^5^. The predominant pathogens were gram-positive cocci (GPC) and gram-negative bacilli (GNB); among these, the streptococcal species were the most common^5^.

However, The Eye Bank Association of America (EBAA) has recently reported an increasing trend in the incidence of fungal infection after corneal transplantation^8^. Given the improvement in prophylactic antibiotics, aseptic procedures, surgical techniques, and postoperative care protocols since our previous study 20 years ago, we here aimed to reexamine the clinical profiles of post-PK infectious keratitis between 2009 and 2014, at the same university hospital. We compared the results of the 2009–2014 period to those of the 1989–1994 study to determine the recent clinical and microbiological profiles, and thereby identify whether trends have changed during the 20-year interval between the studies.

## Methods

This retrospective study was approved by the Institutional Review Board of the National Cheng-Kung University Hospital (NCKUH), which waived the need for informed consent because patient anonymity was maintained. We reviewed the medical records of all patients who underwent corneal transplantations at NCKUH, a tertiary referral center in Taiwan, from November 1, 2009 to October 31, 2014. We excluded patients with lamellar keratoplasty, including DALK or EK, and those who were lost to follow-up for 12 months.

Protocols for medications use after PK have varied widely, and no consensus on optimal methods of administration, dosage, or treatment duration had been achieved^9^. Our post-PK treatment protocol was similar to the other reports, in brief, we used topical prednisolone 1% combined with topical moxifloxacin four times daily. Topical or systemic cyclosporine was not administered. Typically, topical moxifloxacin was tapered over a period of a few months, and prednisolone 1% was tapered down over 3 months with a change to 0.1% fluorometholone four times daily life long^9^,^10^. As for the suture removal, our protocol was also consistent with previous reports^10^,^11^. Sutures were generally removed between 12 and 24 months after surgery. Earlier suture removals were performed in cases of loosening stitches, increased vascularizations, and the presence of infiltrations.

Infectious keratitis was diagnosed based on progressive corneal infiltration and signs of ocular inflammation. At an early stage of the graft infection, thorough laboratory examinations were conducted, including Gram and acid-fast stains, culturing of samples scraped from the active corneal lesion on blood, chocolate, and Sabouraud’s dextrose agar, in thioglycolate broth, and in Lowenstein–Jensen agar slant. We recorded and analyzed the patient age and sex, indications for PK, the time interval between PK and onset of infection, predisposing risk factors, and pathogens identified in the infected grafts.

Categorical variables were analyzed using the chi-square or Fisher’s exact test. Continuous variables were analyzed using Student’s *t* test. A P value < 0.05 was considered as statistically significant. All statistical analyses were performed in SPSS software, version 17 (IBM, Armonk, NY, USA).

## Results

### Demographics

From November 1, 2009 to October 31, 2014, there were 197 corneal transplantations performed. The donor corneas were obtained domestically and from abroad. The average age of the donors was 58.9 ± 14.0 years, M/F ratio 1.4:1, and the elapsed time till preservation was 7.6 ± 2.0 hours. We excluded those cases who underwent EK (n = 40) or DALK (n = 6), and patients who were lost to follow-up for 12 months (n = 13). Finally, 138 patients who underwent PK were included. Demographic data and clinical features are summarized in Table 1. Nine infected eyes of 9 patients were identified among the 138 PK cases during 2009–2014. The mean age of the patients with infectious keratitis was 74.1 ± 12.4 years (range: 55–92 years).The overall infection rate decreased from 11.6% (41 of 354 cases during 1989–1994) to 6.5% (9 of 138 cases during 2009–2014). The age and sex distribution of the infected patients were similar between the 2 study periods (P = 0.100, P = 0.464, respectively).

### Onset of post-PK infectious keratitis

**Figure 1:**
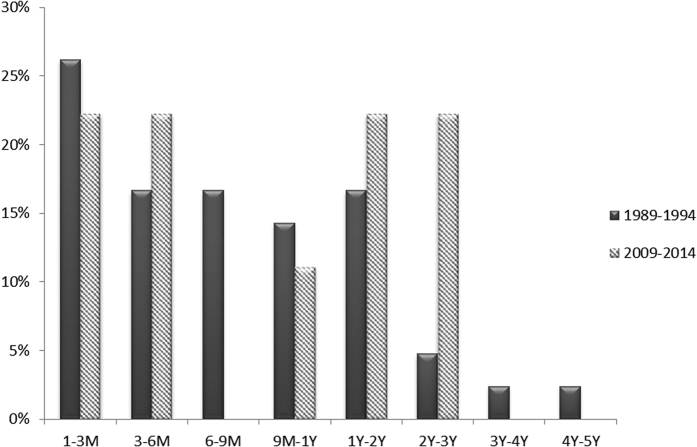
Onset of infection in 37 patients (41 eyes) in 1989–1994 versus the 9 patients (9 eyes) in 2009–2014 who developed post-PK infectious keratitis.

Since post-PK microbial keratitis can occur at variable times, we also evaluated whether the time interval between PK and infection had changed over the past 20 years. The mean time interval of PK to graft infection during 1989–1994 was 10.4 ± 10.9 months, and was 12.8 ± 10.8 during 2009–2014 (P = 0.562; Fig. 1). Overall, 73.8% graft infection episodes occurred in the first year after PK during 1989–1994, as compared to 44.4% during 2009–2014. There was no significant difference between the 2 study periods.

### Indications and predisposing factors for post-PK infectious keratitis

The indications for post-PK infectious keratitis during 2009–2014 are shown in Table 2, and including regrafting (44.4%), impending or perforated corneal ulcer (11.1%), corneal scarring (22.2%), and pseudophakic bullous keratopathy (PBK, 22.2%). Only the frequency of regrafting (P = 0.009) had significantly increased during 2009–2014 as compared to 1989–1994.

The predisposing factors of post-PK infectious keratitis in 1989–1994 and 2009–2014 are summarized in Table 3. Epithelial defects remained the leading risk factor. All of the nine infected grafts were sutured by 10/0 nylon interrupted sutures. None of the other predisposing factors, such as suture-related problems, contact lens use, trichiasis, dry eye, and lid abnormalities showed any difference over the 20-year period.

### Organisms isolated in post-PK infectious keratitis

However, the organisms isolated from the infected corneal grafts during 1989–1994 and 2009–2014 varied markedly (Table 4). GPC and GNB infection after PK decreased from 58.5% and 46.3% to 11.1% and 22.2%, respectively (P = 0.023 and P = 0.271, respectively). In contrast, fungal infections increased from 9.8% to 66.7% (P = 0.001), and fungi are now the major pathogen involved in infections after PK. The overall incidence of fungal keratitis also significantly increased from 1.1% during 1989–1994 to 4.3% during 2009–2014 (P = 0.033). In the recent study period, 4 grafts regained clarities after treatment; the remaining 5 grafts failed. Timely diagnosis will improve the prognosis, especially for the *Candida* species.

## Discussion

Post-PK infectious keratitis is recognized as a major complication and can lead to graft failure^12^. It is generally accepted that there are regional variations in the risk factors and microorganisms involved in post-PK infectious keratitis^13^. In this study, we used identical criteria to select patients from the same university hospital during 2 distinct periods, to identify the changing trends in post-PK infectious keratitis. We found that the overall post-PK infectious keratitis incidence decreased from 11.6% (1989–2004) to 6.5% (2009–2014). Although not statistically significant, we consider that the lower incidence is related to the improvement in surgical instruments and post-operative care protocols. As for the indications of post-PK infections keratitis, unsurprisingly, the regrafting cases significantly increased (P = 0.009) from 5.4% (1989–1994) to 44.4% (200–2014), which was compatible with the shifting trend observed in the United States^14^.

Although the predisposing factors and the onset of post-PK infectious keratitis had not changed in the past 2 decades, the microbiological profiles were significantly different. In previous studies, GPC, including coagulase-negative staphylococci, *Staphylococcus aureus*, *Streptococcus pneumoniae*, viridans streptococci, and other alpha-hemolytic streptococci, were the pathogens most commonly isolated from patients with post-PK infections^5^,^7^. GNB, mainly *Pseudomonas aeruginosa*, was identified as another leading cause^5^,^7^. In the past few decades, fungal species only accounted for 2.8–18.8% of microbial keratitis occurring after PK^15^,^16^,^17^. Our previous data also showed that fungal species accounted for only 9.8% of infectious keratitis after PK during 1989–1994^5^. However, in the present study of the period 2009–2014, the incidence significantly increased (P = 0.001) to 66.6%. Our result was consistent with the most recent data from the online adverse reaction reporting system presented to the Medical Advisory Board of the EBAA in June 2015, which confirmed that the incidence of post-keratoplasty fungal keratitis has increased 4-fold from 2010 to 2014. Similarly, Sung and colleagues reported a frequency of 39.3% of fungal keratitis after PK during 2005 to 2013^13^, and also showed a relatively higher incidence of graft fungus infection. Sung *et al*^13^. mentioned that a considerable number of patients in their study were agricultural workers, which is thought to be an important risk factor for fungal infection. However, in our study, farming was not the major occupation in our post-PK fungal keratitis group, but we found that more than half of our patients lived in suburban areas, which may lead to a higher chance of fungal exposure.

Since our post-PK care protocol for steroid use had not changed in the past 2 decades, we considered that there might be several reasons for the increase in post-PK fungal infection. First, unlike the organ culture method used in Europe, which involves addition of anti-fungal agents, we used Optisol-GS to store the corneas. In the recent study period (2009–2014), one of the nine infected cases (1/9, 11.1%) revealed positive culture result (*Candida albicans*) from the Optisol-GS. This was similar to the previous report, which revealed a 14% incidence of positive donor corneoscleral rim cultures in recipients of postkeratoplasty fungal infection^18^. Although there have been discussions about whether anti-fungal agents should be added to Optisol-GS, there has been no consensus to date^19^. Therefore, the lack of protection against fungi may be a reason for the higher incidence of fungal infection postoperatively. Second, we considered that the improvement of commercialized topical antibacterials played an important role in the changing trend in the isolated microorganisms. During 2009–2014, our team has routinely applied moxifloxacin 4 times per day as a post-operative protocol. The use of broad-spectrum topical antibiotics has significantly declined the prevalence of post-PK bacterial keratitis. However, aggressive use of broad-spectrum topical antibiotics may eradicate both pathological and normal bacterial flora on the ocular surface. In some critically ill patients in the intensive care unit or terminal stage cancer patients, systemic broad-spectrum antibiotics use is considered as a risk factor for fungal infection^20^,^21^. The normal bacterial flora can suppress colonization by other pathogens, such as fungal species^22^. Thus, we surmise that eradication of the normal flora by broad-spectrum topical antibiotic use, combined with the graft epithelial defect and topical steroid usage, would encourage the proliferation of fungal species and increased the opportunistic fungal infection incidence.

In recent years, several studies have shown a changing trend in microbial keratitis over the past 20 years. Lalitha *et al*. found that, although bacterial keratitis was more common than fungal keratitis early in their study period, fungal keratitis became more common after 2004 and the proportion of fungal keratitis steadily increased over time^23^. This preponderance of fungal corneal infections is consistent with some recent studies from other sites in tropical locations, including South Asia and China^24^,^25^,^26^,^27^,^28^,^29^,^30^; however, the reason for the decline in bacterial keratitis is undetermined.

There are a few limitations in this study. First, there were only 138 PK cases during 2009–2014, which was less than the 354 PK cases seen during 1989–1994. The fewer cases of PK during 2009–2014 may be attributable to the improvement in treatment for keratoconus, corneal ulcer, and PBK, which may have decreased the need for corneal transplantation. Moreover, almost all patients with endothelium dystrophy received EK and patients with keratoconus received DALK after 2010. Such a shifting trend in corneal transplantation was also noted recently by Bidaut-Garnier *et al*^31^. Second, the differences in topical antibiotics used for post-PK care may have contributed to the lower bacterial infection rate in the current study period. However, since the overall infection rate decreased, despite the increasing incidence of fungal infection noted in the current study, use of broad-spectrum topical antibiotics is still mandatory.

In conclusion, while the overall post-PK infectious keratitis incidence has decreased over time, fungal infection after PK has increased significantly over the past 2 decades at a university hospital in Taiwan. Our result adds to the current observation from the EBAA, and clinicians should be aware of the shifting trend in pathogens involved in post-PK infectious keratitis. More studies are required to determine whether prophylactic antifungal agents should be added to Optisol-GS or should be used in post-PK patients.

## Additional Information

**How to cite this article**: Lin, I.-H. *et al*. A comparative, retrospective, observational study of the clinical and microbiological profiles of post-penetrating keratoplasty keratitis. *Sci. Rep.* **6**, 32751; doi: 10.1038/srep32751 (2016).

## Figures and Tables

**Table 1:** Demographics of the 37 patients (41 eyes) in 1989–1994 versus the 9 patients (9 eyes) in 2009–2014 developing infectious keratitis after PK.

	1989–1994 (n = 37)	2009–2014 (n = 9)	*P* value
Age, mean (SD), years (range)	66.2 (8.4) (47–80)	74.1 (12.4) (55–92)	0.100
Sex			
Male	19	3	0.464
Female	18	6	0.464
Incidence of infectious keratitis after PK	41/354 (11.6%)	9/138 (6.5%)	0.100
Onset of PK and infectious keratitis, mean(SD), months (range)	10.4 (10.9) (1–52)	12.8 (10.8) (1–31)	0.562

**Table 2:** Indications of post PK infectious keratitis patients in 1989–1994 versus 2009–2014.

Indications of PK	No. of patients (%)		*P* value
	1989–1994 (n = 37)	2009–2014 (n = 9)	
Corneal scarring	21 (56.7)	2 (22.2)	0.135
Impending or perforated corneal ulcer	9 (24.3)	1 (11.1)	0.659
Pseudophakic bullous keratopathy	5 (13.6)	2 (22.2)	0.609
Regrafting	2 (5.4)	4 (44.4)	0.009

**Table 3:** Predisposing factors of infectious keratitis after PK in the 41 eyes in 1989–1994 and 9 eyes in 2009–2014.

Predisposing factor	No. of eyes (%)*		*P* value
	1989–1994 (n = 41)	2009–2014 (n = 9)	
Epithelial defects	20 (48.8)	7 (77.8)	0.270
Suture-related problems	17 (41.5)	3 (33.3)	0.711
Contact lens use	7 (17.1)	0 (0)	0.316
Trichiasis	7 (17.1)	0 (0)	0.316
Dry eye	1 (8.3)	2 (22.2)	0.093
Lid abnormalities	4 (9.7)	1 (11.1)	1.000

^*^Total is greater than 100% because of co-existent factors.

**Table 4:** Organisms Isolated from post PK infectious keratitis during 1989–1994 and 2009–2014.

Organism	No. of eyes (%)		*P* value
	1989–1994 (n = 41)*	2009–2014 (n = 9)	
Gram-positive cocci	24 (58.5)	1 (11.1)	0.023
*Staphylococcus aureus*	3 (7.3)	1 (11.1)	
Gram-negative bacilli	19 (46.3)	2 (22.2)	0.271
*Pseudomonas aeruglnosa*	4 (9.8)	2 (22.2)	
Fungi	4 (9.8)	6 (66.7)	0.001
*Candida ablicans*	2 (4.9)	1 (7.7)	
*Candida parapsilosis*	2 (4.9)	0 (0)	
*Candida glabrata*	0 (0)	1 (7.7)	
*Candida tropicalis*	0 (0)	1 (7.7)	
*Scedosporium spp.*	0 (0)	1 (7.7)	
*Paecilomyces spp.*	0 (0)	1 (7.7)	
*Penicillium spp.*	0 (0)	1 (7.7)	

^*^Total is not 100% because some infections involved more than one organism.
